# Rapid Interventions to Limit Outbreak of Invasive *Streptococcus pneumoniae* in Correctional Facility, North Carolina, USA, 2024

**DOI:** 10.3201/eid3203.250789

**Published:** 2026-03

**Authors:** Camden D. Gowler, Emma Doran, Niketa D. Williams, Justin P. Albertson, Ty Lautenschlager, Sopio Chochua, Ryan Gierke, Arthur Campbell, Miwako Kobayashi, Aaron Fleischauer, Erica Wilson

**Affiliations:** Centers for Disease Control and Prevention, Atlanta, Georgia, USA (C.D. Gowler, S. Chochua, R. Gierke, M. Kobayashi, A. Fleischauer); North Carolina Department of Health and Human Services, Raleigh, North Carolina, USA (C.D. Gowler, E. Doran, N.D. Williams, J.P. Albertson, T. Lautenschlager, A. Fleischauer, E. Wilson); North Carolina Department of Adult Correction, Raleigh (A. Campbell)

**Keywords:** Streptococcus pneumoniae, bacteria, streptococci, correctional facility, vaccines, serotyping, outbreak, pneumococcal disease, pneumococcus, pneumococcal disease, PPSV23, North Carolina, United States

## Abstract

A *Streptococcus pneumoniae* serotype 4 outbreak in a North Carolina correctional facility resulted in 14 cases (8 suspected, 1 probable, and 5 confirmed). After implementation of movement restrictions and vaccination with 23-valent pneumococcal polysaccharide vaccine, new cases ceased. Serotype 4 presence in this setting raises challenges for an effective vaccination strategy.

*Streptococcus pneumoniae* (pneumococcus) is a leading bacterial cause of community-acquired pneumonia in the United States (*1*). Although infrequent, pneumococcus can also cause invasive pneumococcal disease (IPD), an infection in a normally sterile site (i.e., blood, cerebrospinal fluid, or bone or joint space) (*2*). Young children, persons with certain underlying conditions or risk factors (e.g., chronic heart, liver, or lung disease; HIV infection; cigarette smoking), and older adults are at increased risk for IPD (*1*).

IPD outbreaks can occur in congregate settings, such as nursing homes or correctional facilities (i.e., jails and prisons), and result in substantial illness and death (*3*,*4*). Close living quarters increase risk for pneumococcal transmission. Effective vaccines against IPD are available (*5*); however, pneumococcal vaccines confer protection only to the specific *S. pneumoniae* serotypes contained in vaccines (*5*). That limitation poses a challenge to timely use of vaccines for containing outbreaks because delays in determining pneumococcal serotypes often occur. Emergence of serotype 4 IPD cases among certain adult populations might further complicate vaccine product selection (*6*,*7*). Newer pneumococcal conjugate vaccines (PCVs) cover more serotypes, but the 21-valent PCV (PCV21) that was most recently recommended for adults in 2024 (*5*) does not contain serotype 4, whereas other recommended pneumococcal vaccines do. As of 2023, serotype 4 was uncommon in the southeastern United States, according to available data from the Centers for Disease Control and Prevention (CDC) Active Bacterial Core surveillance (*8*).

On June 26, 2024, the North Carolina Division of Public Health was informed of multiple pneumonia and pneumococcal disease cases at a minimum custody correctional facility in North Carolina, USA. We conducted an investigation to characterize cases identified within the facility during June 14–July 30, 2024, and to determine intervention strategies.

## The Investigation

We classified cases into 3 categories: suspected, probable, and confirmed (Appendix). Suspected cases were defined as new-onset respiratory symptoms necessitating antibiotic treatment but without laboratory-confirmed pneumococcal infection. Probable cases were defined as radiograph-confirmed pneumonia or clinical or laboratory signs of sterile site infection (e.g., sepsis) without pneumococcal detection. Confirmed cases were defined as culture-confirmed *S. pneumoniae* from a normally sterile site. Pneumococcal isolates were sent to CDC for serotyping and to assess genetic relatedness by single-nucleotide polymorphism analysis. This activity was reviewed by CDC, deemed not research, and was conducted consistent with applicable federal law and CDC policy (see, e.g., 45 C.F.R. part 46.102(l) (*2*), 21 C.F.R. part 56; 42 U.S.C. §241(d); 5 U.S.C. §552a; 44 U.S.C. §3501 et seq.).

We identified 14 cases (8 suspected, 1 probable, and 5 confirmed) among 267 incarcerated persons (Table; Figure). Persons with suspected cases had mild respiratory symptoms (i.e., cough); those with probable and confirmed cases had more severe signs and symptoms (Table). All persons with confirmed and probable cases were hospitalized, and all survived (Table). Median age at illness onset was 51 (range 29–68) years. Most (79%, n = 11) cases, including all confirmed and probable cases, occurred among Black men. Among confirmed and probable cases, 4 (67%) of 6 occurred among current or former smokers. Most (79%, n = 11) cases occurred among persons with occupations while incarcerated, either within the facility (e.g., kitchen or custodial staff) or as part of work-release (e.g., nearby poultry plant). We abstracted pneumococcal vaccination statuses for patients from medical records and compared those records with the North Carolina Immunization Registry. No patient had a documented pneumococcal vaccination history.

**Table:** Demographics and risk factors of pneumococcal infections by case classification among patients in correctional facility, North Carolina, USA, June 14–July 30, 2024*

Category	Confirmed	Probable	Suspected
Total no. cases	5	1	8
Median patient age, y (range)	42 (39–62)	29 (NA)	54.5 (30–68)
Race			
Black or African American	5 (100)	1 (100)	5 (62.5)
White	0	0	3 (37.5)
Work status			
None	1 (20)	0	2 (25)
Poultry plant	2 (40)	0	1 (12.5)
Other work†	2 (40)	1 (100)	5 (62.5)
Current	2 (40)	0	NA
Smoking status‡			
Former	2 (40)	0	NA
Never	1 (20)	1 (100)	NA
Select signs and symptoms			
Fever	2 (40)	1 (100)	0
Cough	4 (80)	1 (100)	8 (100)
Radiograph-confirmed pneumonia	5 (100)	1 (100)	0
Clinical signs of sepsis	4 (80)	1 (100)	0
Lumbar puncture–confirmed meningitis	1 (20)	0	0
Testing and treatment			
Treated with antibiotics§	5 (100)	1 (100)	8 (100)
Positive culture from normally sterile site	5 (100)	0	0
Hospitalized	5 (100)	1 (100)	0

*Values are no. (%) except as indicated. NA, not applicable. †Other work includes custodial staff, kitchen, and municipal worker (any job except at the poultry plant), some of which occurred outside the correctional facility. ‡Smoking status was determined by medical record review. Records were only available to sufficiently review confirmed and probable cases. §Empiric treatment with antibiotics only. Postexposure antibiotic prophylaxis was not administered, but providers might have had a higher suspicion of pneumococcal disease after the outbreak began.

**Figure:**
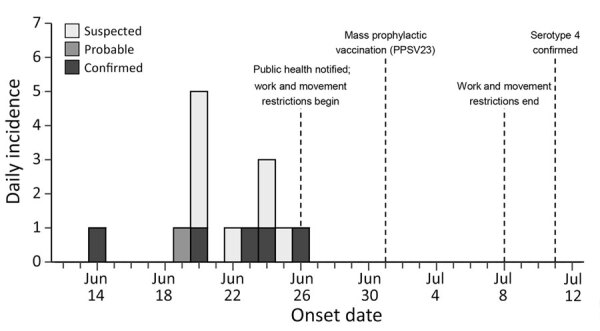
Incidence of pneumococcal disease by day and case classification among patients in correctional facility, North Carolina, USA, June 14–July 30, 2024. Vertical dashed lines and labels denote key events during the public health investigation and response. PPSV23, 23-valent pneumococcal polysaccharide vaccine.

All incarcerated persons and facility staff were offered prophylactic vaccination with 23-valent pneumococcal polysaccharide vaccine (PPSV23); PPSV23 was most readily available for purchase in sufficient quantities. On July 1, 2024, five days after the outbreak was reported, multiple staff and 157 (59%) incarcerated persons received PPSV23. Postexposure antibiotic prophylaxis was not offered, but symptomatic persons received empiric antibiotic treatment with azithromycin. Work-release and movement within the correctional facility were restricted from June 26 through July 8, 2024. No additional cases were reported after June 26, 2024.

Pneumococcal isolates from laboratory-confirmed cases were all serotype 4, multilocus sequence type (ST) 695. The average core-genome single-nucleotide polymorphism difference was 10, indicating isolates were closely related. All 5 isolates were pansusceptible to relevant antibiotics (penicillin, amoxicillin, cefotaxime, ceftriaxone, cefuroxime, meropenem, vancomycin, erythromycin, tetracycline, doxycycline, levofloxacin, trimethoprim/sulfamethoxazole, chloramphenicol, rifampin, clindamycin, quinupristin/dalfopristin, and linezolid) on the basis of whole-genome sequencing predictions (*9*).

This IPD serotype 4 outbreak occurred in a correctional facility located in a geographic area where serotype 4 is considered to be uncommon. IPD outbreaks in congregate settings, such as correctional facilities, remain a public health concern. Rapid detection of the outbreak and timely interventions were aided by collection of bacterial cultures. In this outbreak, before serotyping results were available, public health officials chose PPSV23 on the basis of availability; fortunately, that vaccine covered the outbreak strain (serotype 4).

Historically, serotype 4 IPD cases declined across all ages after routine pneumococcal conjugate vaccine (PCV) use in children began in 2000 (*10*). Since 2013, serotype 4 IPD cases in the western United States have increased among adults; clusters of IPD cases were observed among persons experiencing homelessness and among adults with underlying conditions or risk factors, including injection drug use (*8*,*9*). The increase in serotype 4 invasive IPD in the western United States has been associated with lineages ST10172, ST244, and ST695, with ST10172 identified as most prevalent (*7*). Recent CDC Active Bacterial Core surveillance data indicate a notable decline in the serotype 4/ST695 prevalence since 2015 (CDC, unpub. data), largely supplanted by ST10172.

This outbreak response underscores continued risk for IPD outbreaks within correctional facilities, where close quarters and underlying risk factors for IPD (e.g., history of cigarette smoking or chronic medical conditions) can be common (*3*,*10*,*11*). Although public health studies of correctional facilities are limited (*12*), the most recently described US correctional facility IPD outbreak occurred in an Alabama state prison in 2018 but was caused by serotype 12F (*3*). 

## Conclusions

Assessing large, mobile populations, such as the population of the United States, for asymptomatic carriage of *S. pneumoniae* serotypes is difficult because of the large number of persons required to test; a study in Europe reported prevalence among adult men is ≈3.7% (*13*). Our report indicates serotype 4 IPD clusters are not geographically restricted to the western United States. Given the potential increased risk in correctional facilities, coupled with the absence of documented pneumococcal vaccination among persons in this outbreak, officials might consider serotyping IPD to guide choice of vaccine in similar outbreaks.

In addition to work-release and movement restrictions, a primary public health intervention in this response was prophylactic vaccination with PPSV23. Antibiotic prophylaxis was not administered because determining close contacts for prophylaxis would have been logistically challenging and could have included >200 incarcerated persons. Prophylactic vaccination with PPSV23 appeared successful; rollout was fast and covered the outbreak strain (serotype 4), and no cases were documented after vaccination in the correctional facility. Although conjugate vaccines (PCV15, PCV20, PCV21) are more immunogenic than polysaccharide vaccines (PPSV23), timely administration of PPSV23 might have prevented additional serotype 4 IPD cases.

Without serotype results, public health officials might have opted to use PCV21, had it been available. Compared with other vaccines, PCV21 is thought to cover more circulating serotypes in the eastern United States, and surveillance data did not indicate a high probability of serotype 4 causing an outbreak in North Carolina (*14*). Serotyping may be obtained through state public health laboratories or CDC, subject to availability. Rapid serotyping of pneumococcal cases can inform vaccine selection in managing IPD within specific environments, including correctional facilities.

Appendix Additional information for rapid interventions to limit outbreak of invasive *Streptococcus pneumoniae* in correctional facility, North Carolina, USA, 2024.
