# Supplementary material for: Rapid Interventions to Limit Outbreak of Invasive Streptococcus pneumoniae in Correctional Facility, North Carolina, USA, 2024
Source: Emerg Infect Dis. 2026 Mar;32(3):424–7. doi: 10.3201/eid3203.250789 (PMC13016010; doi:10.3201/eid3203.250789)
Supplement: Appendix — Additional information for rapid interventions to limit outbreak of invasive Streptococcus pneumoniae in correctional facility, North Carolina, USA, 2024. [file 25-0789-Techapp-s1.pdf]

Article DOI: https://doi.org/10.3201/eid3203.250789

EID cannot ensure accessibility for supplementary materials supplied by authors. Readers who have difficulty accessing supplementary content should contact the authors for assistance.

Rapid Interventions to Limit Outbreak of Invasive *Streptococcus pneumoniae* in Correctional Facility, North Carolina, USA, 2024

Appendix

Case Definitions for Pneumococcal Disease

Case Definitions: Demonstrating the following signs and symptoms on or after June 14, 2024 (and up to 1 month after last case identification), in a person incarcerated in the correctional facility of interest or who has had prolonged close contact with a person who is incarcerated there.

1. Suspected pneumococcal case
   a. Suspected pneumonia
      i. Pneumonia diagnosed by a clinician, but no radiographic studies or radiographs were negative.

   OR

   b. Symptomatic respiratory infection
      i. Treatment with antibiotics and any of the following, without meeting the above case definitions:
         1. Fever ≥38°C
         2. Cough
         3. Chest pain
         4. Difficulty breathing

5. Sore throat

6. Ear pain

OR

c. Symptomatic bacterial meningitis

i. Treatment with antibiotics and any of the following, without meeting the above case definitions:

1. Fever ≥38°C

2. Neck stiffness

3. Altered mental status

4. Other meningeal signs

2. Probable pneumococcal case

a. Radiographically confirmed pneumonia

OR

b. Clinical or laboratory signs of sterile site infection without detection of *Streptococcus pneumoniae* (as described in confirmed case criteria)

i. Sepsis diagnosed in medical records

ii. Meningitis diagnosed by lumbar puncture

AND

c. No other bacteria species identified from sterile site

3. Confirmed pneumococcal case

a. Clinical illness compatible with *S. pneumoniae*

i. Radiograph-confirmed pneumonia OR

ii. Signs of sterile site infection (e.g., bacteremia, sepsis, or meningitis)

AND

b. *S. pneumoniae* isolated from a normally sterile site (e.g., blood, cerebrospinal fluid, or pleural fluid)
